# Optimal cutoff values for visceral fat volume to predict metabolic syndrome in a Korean population

**DOI:** 10.1097/MD.0000000000027114

**Published:** 2021-09-10

**Authors:** Yun-A Kim, Sang Gyu Kwak, Yoon Jeong Cho

**Affiliations:** a Department of Family Medicine, Daegu Catholic University School of Medicine, Daegu, Republic of Korea; b Department of Medical Statistics, Daegu Catholic University School of Medicine, Daegu, Republic of Korea.

**Keywords:** fat volume, metabolic syndrome, subcutaneous fat, visceral fat

## Abstract

Previous studies have reported the association between visceral fat and metabolic syndrome (MS); however, just few studies have been conducted to evaluate the relationship between actual visceral fat volume (VFV) and MS. This study aimed to obtain 3 dimensional VFV and subcutaneous fat volume (SFV) using abdominal computed tomography (CT) and determine MS-predictive cutoff values.

A total of 250 individuals, aged 27 to 80 years, who underwent health screening with abdominal CT between November 2019 and May 2020 were included. The subcutaneous (SFA) and visceral (VFA) fat areas were quantified using axial images obtained at the level of the lowest to the highest part of the umbilicus. The SFV and VFV were quantified from the highest level of the liver dome to the pelvic floor on axial CT images. The Aquarius iNtuition software program (TeraRecon, Foster City, CA) was used to calculate the SFA, VFA, SFV, and VFV. Subcutaneous fat mass and visceral fat mass (VFM) were measured using an adipose tissue density of 0.9 g/mL. We used the modified criteria of MS proposed by the Third National Cholesterol Education Program Expert Panel on Detection, Evaluation, and Treatment of High Blood Cholesterol in Adults and waist circumference of ≥90 cm in men and ≥85 cm in women to define MS. Multivariate analysis of covariance was used to compare the fat areas, volumes, and mass according to the presence of MS and sex. Additionally, a receiver operating characteristic curve analysis was performed to determine the cutoff values for VFV, SFV, VFM, subcutaneous fat mass, VFA, and SFA associated with MS.

Of the assessed variables, VFV and VFM had the highest area under the receiver operating characteristic curve value for predicting MS in both men and women: 0.811 (95% confidence interval, 0.743–0.868) for men and 0.826 (95% confidence interval, 0.727–0.900) for women. The MS-predictive cutoff values were 4852 cm^3^ and 4366.8 g for men and 3101 cm^3^ and 2790.9 g for women, respectively. Further, large, population-based studies are needed to validate these cutoff values.

## 1 Introduction

Abdominal obesity is an important component of the metabolic syndrome (MS), independent of overall obesity.^[1,2]^ As a result, central fat deposits are a significant part of the pathogenesis of metabolic disorders. Abdominal adipose tissue is divided into visceral and subcutaneous adipose tissues, which have different metabolic risk profiles. The correlation between visceral adipose tissue and cardiometabolic risks is stronger than that between subcutaneous adipose tissue and cardiometabolic risks.^[3–5]^ In addition, the actual visceral adipose tissue volume is a more accurate predictor of MS than the visceral adipose area.^[6]^ Fats are stored in visceral adipose tissue when subcutaneous adipose tissue is saturated.^[4]^ Besides body fat distribution, the small adipocyte size and secretion of inflammatory cytokines in visceral adipose tissues are potential mechanisms of the pathogenic effects of visceral fat on cardiometabolic risk.^[7]^ In addition, the transportation of free fatty acids and inflammatory mediators from visceral adipose tissue to the liver is increased owing to the direct communication between adipose tissues and the liver through the portal circulation.^[8]^ This can lead to insulin resistance, hepatic steatosis, and dyslipidemia.^[9]^

In a recent cohort study with a 9.3-year follow-up, visceral fat increased the risk of cardiovascular disease (CVD) in individuals in the middle and high tertiles of visceral fat. These results were similar after adjustment for body mass index (BMI). However, there was no significant association between subcutaneous fat and CVD events.^[10]^ Moreover, several epidemiological and longitudinal studies have reported an association between glucose intolerance and visceral fat.^[11–13]^ Most existing studies have shown a stronger relationship between visceral adipose tissue and MS than between subcutaneous adipose tissue and MS; however, few studies have yet assessed the relationship between the actual visceral fat volume (VFV) and MS.

Computed tomography (CT) and magnetic resonance imaging (MRI) can directly measure the areas or volumes of adipose tissue: they are reference methods for evaluating abdominal adiposity.^[14]^ CT and MRI have been used to investigate the associations between actual adipose tissue volume and other anthropometric indices.^[4,15]^ MRI-derived adipose tissue area is a reported accurate predictor of MS, with a stronger association with MS than the association of BMI with MS.^[16]^ However, MRI is more expensive and time-consuming than CT.^[17]^ In several studies, specific single axial CT images have been used to measure abdominal adiposity because of its simplicity and associated reduced radiation exposure.^[10,18–20]^ Because the diagnostic criteria for abdominal obesity are based on the visceral fat area (VFA), VFA measurements using single axial CT are required.^[21]^ However, single-slice imaging may not be as accurate as total volume imaging in detecting longitudinal changes in abdominal adiposity.^[14,15]^ Few validation studies have shown that measuring VFV using CT is feasible and highly accurate.^[15,17]^ However, there is a paucity of studies on the associations between the actual fat volume and cardiometabolic diseases.

In this study, we aimed to evaluate the association of estimated VFV and subcutaneous fat volume (SVF) quantified using abdominal CT which is less expensive and needs less time to get images compared to MRI along with other obesity parameters and to investigate the appropriate cutoff values for fat volumes to predict MS.

## 2 Methods

### 2.1 Data collection and study participants

This study was based on the data acquired during health screening tests conducted at a university hospital located in the Republic of Korea between November 2019 and May 2020. We included participants who underwent abdominal CT. A total of 250 individuals aged 27 to 80 years were included in the analyses. The study protocol was approved by the Institutional Review Board of Daegu Catholic University Medical Center (IRB approval number, CR-20-046).

### 2.2 Determination of areas, volumes, and mass of subcutaneous fat and visceral fat on CT images

The subcutaneous fat area (SFA), VFA, SFV, and VFV were quantified using the Aquarius iNtuition software program (TeraRecon, Foster City, CA). We used a tissue phantom to validate the Hounsfield Unit (HU) range for identifying adipose tissues (−150 to −50 HUs for visceral adipose tissue, −190 to −30 HUs for subcutaneous adipose tissue).^[22]^ SFA and VFA were quantified using axial images obtained at the level of the lowest to the highest part of the umbilicus and near the L4 to L5 vertebral interspace. SFV and VFV were quantified from the highest level of the liver dome to the pelvic floor and the highest level of the anal sphincter on axial CT images.^[6,15,23]^ These measurements were performed by the same examiner to prevent inter-observer variability. Subcutaneous (SFM) and visceral (VFM) fat mass were quantified using an adipose tissue density of 0.9 g/mL.

### 2.3 Definition of MS

We used the modified criteria of MS proposed by the Third National Cholesterol Education Program Expert Panel on Detection, Evaluation, and Treatment of High Blood Cholesterol in Adults^[2]^ and the specific waist circumference (WC) values for Koreans suggested by the Korean Society for the Study of Obesity to define MS.^[24]^ MS was diagnosed when 3 or more abnormal findings among 5 risk factors were present.^[25]^ The risk factors included WC ≥90 cm in men and ≥85 cm in women; systolic blood pressure (SBP) ≥30 mm Hg, diastolic blood pressure (DBP) ≥85 mm Hg, or previous diagnosis of hypertension with antihypertensive drug treatment; fasting blood glucose (FBG) level ≥100 mg/dL or previous diagnosis of diabetes with drug treatment; triglyceride (TG) level ≥150 mg/dL; and high-density lipoprotein cholesterol (HDL-C) level <40 mg/dL for men and <50 mg/dL for women.

### 2.4 Assessment of lifestyle habits and comorbidities

Lifestyle habits and comorbidities were assessed using a self-administered questionnaire. Physical activity was categorized as none, low-intensity, and moderate-to-vigorous intensity exercise based on the average intensity and frequency of exercise within the past year. The none group comprised participants who responded “none” to the question about strenuous exercise (20 minutes a day) or walking (more than 30 minutes a day). The moderate-to-vigorous intensity exercise group comprised participants who reported doing strenuous exercise for 3 or more days or moderate intensity exercise for more than 5 days per week. The low-intensity group comprised participants who were included in neither group.

Regarding smoking, individuals who had never smoked or smoked fewer than 100 cigarettes in their lifetime were classified as nonsmokers. The participants who reported smoking in the past but who had quit smoking were classified as ex-smokers, and those who were smoking at the time of the study were classified as current smokers.

High-risk alcohol consumption was defined as drinking alcohol more than twice per week, with an average of 7 or more glasses for men and 5 or more glasses for women at any time.

Furthermore, the presence of comorbidities such as hypertension, diabetes, dyslipidemia, angina, stroke, fatty liver, and cancer was assessed. In women, menopausal status was assessed based on the self-reported answer to the question about having regular menstrual periods.

### 2.5 Anthropometric measurements and blood tests

Anthropometric measurements were performed during the health checkup, and the participants had to wear the same clothing each time. Height and weight were measured using a standard method to the nearest 0.1 cm for height and 0.1 kg for weight. WC was measured at the midpoint between the lowest level of the ribs and the iliac crest. BMI was calculated by dividing weight in kilograms by height in meters squared. Blood pressure was measured using an automatic sphygmomanometer on the right arm with the patients in a sitting position after 5 minutes of rest. The average SBP and DBP were obtained after repeated measurements.

Blood samples were obtained in the morning after overnight fasting. Moreover, FBG, hemoglobin A1C level, and lipid profiles were assessed.

### 2.6 Statistical analyses

All analyses were performed using IBM SPSS version 19.0 (IBM Co., Armonk, NY). The chi-square test and one-way analysis of variance were used to compare the baseline characteristics of the participants. Continuous variables are expressed as means and standard deviation (SD), and discrete variables are expressed as numbers with proportions. A simple linear regression analysis was performed to examine the correlation between fat volumes and metabolic risk factors. Multivariate analysis of covariance was used to compare the fat areas, volumes, and mass, according to the presence of MS and sex. We adjusted for age, smoking, alcohol consumption, physical activity, and comorbidities to perform independent comparisons of visceral fat and subcutaneous fat according to the presence of MS. Adjustment for menopausal status was made only for women. A receiver operating characteristic (ROC) curve analysis was performed to define the cutoff values for VFV, SFV, VFM, SFM, VFA, and SFA associated with MS. MedCalc for Windows version 18.11.3 (MedCalc Software, Ostend, Belgium) was used for the ROC curve analyses. *P*-value < .05 was considered significant.

## 3 Results

### 3.1 Characteristics of study participants

Table 1 shows the baseline characteristics of the study participants according to sex and the presence of MS. A total of 166 men and 84 women were included in the study. Women with MS were older than those without MS, although there was no difference in age in men according to the presence or absence of MS. Obesity parameters, such as body weight, WC, and BMI, VFA, SFA, SFV, and VFV were higher in men with MS than in those without MS. In women, a similar trend was observed, except for VFA and SFA. SBP, FBG, and TG levels were higher in both men and women with MS than in those without. HDL-C levels were lower in men with MS than in those without. There were no differences in physical activity, smoking, and alcohol consumption according to MS in both men and women. Regarding comorbidities, the prevalence of hypertension, diabetes, and dyslipidemia was higher in both men and women with MS. However, there was no significant difference in the prevalence of dyslipidemia among the women. There were no differences in the prevalence of angina, stroke, fatty liver, and cancers according to the presence of MS among the men or women.

**Table 1:** Baseline characteristics of study populations (N = 250).

	Men			Women		
	MS present (N = 47)	MS absent (N = 119)	*P*-value	MS present (N = 11)	MS absent (N = 73)	*P*-value
Age (yr)	54.3 ± 10.2	54.5 ± 9.8	.908	63.3 ± 10.2	55.3 ± 10.1	.017
Height (cm)	170.5 ± 5.7	170.2 ± 6.0	.756	157.2 ± 4.3	157.1 ± 5.4	.968
Weight (kg)	80.6 ± 10.2	70.9 ± 8.6	<.001	64.4 ± 10.3	58.3 ± 8.5	.032
Waist circumference (cm)	92.7 ± 6.5	85.7 ± 5.5	<.001	87.0 ± 5.6	79.7 ± 7.4	.002
Body mass index (kg/m^2^)	27.7 ± 2.6	24.4 ± 2.2	<.001	26.0 ± 3.0	23.6 ± 2.8	.010
Visceral fat area (cm^2^)	166.2 ± 40.5	115.8 ± 46.3	<.001	112.2 ± 25.4	105.9 ± 229.6	.928
Subcutaneous fat area (cm^2^)	175.8 ± 69.8	130.8 ± 49.4	<.001	193.4 ± 61.9	163.6 ± 60.0	.130
Visceral area ratio	49.8 ± 11.7	47.2 ± 12.0	.202	37.8 ± 9.4	32.8 ± 11.8	.180
Visceral fat volume (cm^3^)	5369.6 ± 1100	3772.3 ± 1400	<.001	3904.6 ± 707.0	2695.4 ± 1600	.014
Subcutaneous fat volume (cm^3^)	5032.3 ± 2000	3501.0 ± 1300	<.001	6716.1 ± 2000	5050.5 ± 1900	.009
Visceral fat volume ratio	52.7 ± 8.3	51.7 ± 7.7	.486	37.7 ± 5.8	33.6 ± 8.2	.122
Systolic blood pressure (mm Hg)	131.4 ± 10.0	125.4 ± 11.2	.002	142.3 ± 14.6	123.5 ± 13.3	<.001
Diastolic blood pressure (mm Hg)	78.9 ± 8.1	78.1 ± 9.6	.631	81.1 ± 12.0	72.2 ± 9.4	.006
Fasting glucose (mg/dL)	115.7 ± 33.0	93.3 ± 13.7	<.001	119.8 ± 25.7	94.0 ± 12.8	.008
Hemoglobin A1C (%)	6.0 ± 1.0	5.5 ± 0.5	.001	6.3 ± 0.9	5.5 ± 0.6	.009
Triglyceride (mg/dL)	186.5 ± 75.7	109.3 ± 61.8	<.001	105.8 ± 45.4	81.2 ± 30.3	.022
HDL cholesterol (mg/dL)	47.5 ± 11.5	57.2 ± 13.7	<.001	56.8 ± 13.9	66.0 ± 15.2	.064
LDL cholesterol (mg/dL)	130.3 ± 45.4	133.1 ± 35.0	.702	107.5 ± 33.2	128.0 ± 32.0	.076
Physical activity, n (%)			.977			.155
None	3 (6.4)	8 (6.7)		3 (27.3)	6 (8.2)	
Low-intensity exercise	29 (61.7)	75 (63.0)		6 (54.5)	48 (65.8)	
Moderate-to-vigorous exercise	15 (31.9)	36 (30.3)		2 (18.2)	19 (26.0)	
Smoking, n (%)			.724			.429
Never smoker	9 (20.0)	23 (20.2)		9 (90.0)	65 (95.6)	
Ex-smoker	21 (46.7)	60 (52.6)		0 (0)	2 (2.9)	
Current smoker	15 (33.3)	31 (27.2)		1 (10.0)	1 (1.5)	
High-risk alcohol intake, n (%)	21 (44.7)	35 (29.4)	.061	1 (9.1)	5 (6.8)	.581
Menopausal state, n (%)	NA	NA		9 (81.8)	2 (18.2)	.500
Comorbidities, n (%)						
Hypertension	20 (42.6)	19 (16.0)	<.001	5 (45.5)	10 (13.7)	.023
Diabetes	13 (27.7)	3 (2.5)	<.001	3 (27.3)	2 (2.7)	.015
Dyslipidemia	15 (31.9)	15 (12.6)	.004	4 (36.4)	11 (15.1)	.102
Angina	3 (6.4)	6 (5.0)	.714	1 (9.1)	0 (0)	.131
Stroke	2 (4.3)	2 (1.7)	.318	1 (9.1)	0 (0)	.131
Fatty liver	1 (2.1)	2 (1.7)	.999	0 (0)	0 (0)	.999
Any cancers	2 (4.3)	6 (5.0)	1.000	0 (0)	3 (4.1)	1.000

### 3.2 Correlations of fat volume and metabolic risk factors with other obesity parameters

Table 2 shows the correlations of SFV and VFV with components of MS and other obesity parameters based on simple linear regression analysis. There was a positive correlation between VFV and all components of MS (SBP, DBP, FBG, serum TG, HDL-C levels, and WC). Among these components, SFV was positively correlated with only SBP and WC, and BMI, SFA, and VFA were positively correlated with both SFV and VFV.

**Table 2:** Correlations of fat volume and metabolic risk factors with other obesity parameters.

	Subcutaneous fat volume	Visceral fat volume
Systolic blood pressure (mm Hg)	0.165^†^	0.252^†^
Diastolic blood pressure (mm Hg)	0.078	0.201^†^
Fasting blood sugar (mg/dL)	0.059	0.240^†^
Serum TG (mg/dL)	0.112	0.464^†^
Serum HDL-C (mg/dL)	−0.100	−0.401^†^
Waist circumference (cm)	0.415^†^	0.740^†^
Body mass index (kg/m^2^)	0.641^†^	0.680^†^
Subcutaneous fat area (cm^2^)	0.881^†^	0.405^†^
Visceral fat area (cm^2^)	0.162^∗^	0.481^†^

### 3.3 Areas, volumes, and mass of body fat according to MS

Table 3 shows the adjusted mean values of fat area, volume, and mass according to the presence of MS and sex, based on multivariate analysis of covariance. Areas, volumes, SFM, and VFM were higher in men with MS than in those without MS. However, there were no significant differences among women.

**Table 3:** Univariate analysis of covariance^∗^ of fat area, volume, and mass according to the presence of metabolic syndrome.

	Men			Women		
	MS present (N = 47)	MS absent (N = 119)	*P*-value	MS present (N = 11)	MS absent (N = 73)	*P*-value
Fat area (cm^2^)						
Subcutaneous fat	180.8 ± 7.7	132.3 ± 4.8	<.001	183.4 ± 21.5	162.1 ± 7.3	.366
Visceral fat	166.2 ± 6.6	118.7 ± 4.1	<.001	95.3 ± 12.7	80.6 ± 4.3	.291
Fat volume (cm^3^)						
Subcutaneous fat	5151.4 ± 205.5	3552.7 ± 128.4	<.001	6164.9 ± 711.5	5035.5 ± 242.4	.150
Visceral fat	5366.5 ± 199.1	3851.0 ± 124.3	<.001	3221.4 ± 471.2	2676.7 ± 160.5	.293
Fat mass (g)						
Subcutaneous fat	4636.3 ± 185.0	3197.4 ± 115.6	<.001	5548.4 ± 640.3	4532.0 ± 218.2	.150
Visceral fat	4829.8 ± 179.2	3465.9 ± 111.9	<.001	2899.3 ± 424.1	2409.0 ± 144.5	.293

### 3.4 Cutoff values of fat areas, volumes, and mass for MS identification

**Figure 1:**
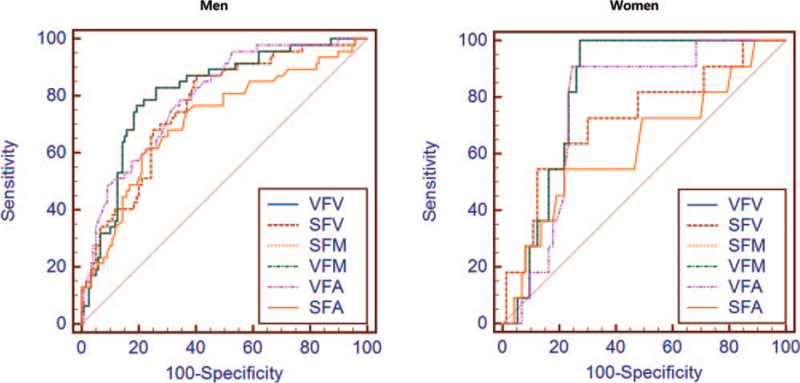
Receiver operating characteristic curves for identifying metabolic syndrome according to sex. VFA = visceral fat area, VFM = visceral fat mass, VFV = visceral fat volume, SFA = subcutaneous fat area, SFM = subcutaneous fat mass, SFV = subcutaneous fat volume.

Figure 1 shows the ROC curves of fat areas, volumes, and mass for identifying MS in men and women. Table 4 presents the area under the ROC curve (AUC) values. All AUC values were above 0.7, except for SFA in women. Among both men and women, the AUCs for VFV, VFM, and VFA were higher than those for SFV, SFM, and SFA. The optimal cutoff values for VFV, SFV, VFM, SFM, VFA, and SFA for the identification of MS are presented in Table 4.

**Table 4:** Cutoff values for fat area, volume, and mass for identifying metabolic syndrome according to sex.

	Men (N = 166)				Women (N = 84)			
	Cutoff	AUC (95% CI)	Sensitivity (%)	Specificity (%)	Cutoff	AUC (95% CI)	Sensitivity (%)	Specificity (%)
VFV (cm^3^)	4852	0.811 (0.743–0.868)	76.6	80.7	3101	0.826 (0.727–0.900)	100	72.6
SFV (cm^3^)	3558	0.764 (0.692–0.827)	87.2	59.7	5860	0.725 (0.616–0.817)	72.7	69.9
VFM (g)	4366.8	0.811 (0.743–0.868)	76.6	80.7	2790.9	0.826 (0.727–0.900)	100	72.6
SFM (g)	3202.2	0.764 (0.692–0.827)	87.2	59.7	5274	0.725 (0.616–0.817)	72.7	69.9
VFA (cm^2^)	138	0.796 (0.727–0.854)	78.7	65.5	95.1	0.768 (0.664–0.853)	90.9	75.3
SFA (cm^2^)	164	0.715 (0.640–0.782)	59.6	79.0	194	0.629 (0.517–0.732)	54.5	78.1

## 4 Discussion

In this study, we demonstrated the optimal cutoff values for fat volume, mass, and area to predict MS by three-dimensional abdominal visceral fat quantification using abdominal CT images. Of the assessed fat parameters, VFV and VFM showed the highest AUC values in both men and women. Because SFM and VFM were calculated using a fat density of 0.9 g/mL, there were no differences in the AUC values between fat mass (SFM and VFM) and volume. The cutoff values of VFV and VFM were 4852 cm^3^ and 4366.8 g and 3101 cm^3^ and 2790.9 g in men and women, respectively.

The association between central obesity and diabetes has been well known. In a 10-year longitudinal study on the development of type 2 diabetes, an increase of 1 SD in the intraabdominal fat area increased the likelihood of type 2 diabetes as much as 1.65 times (odds ratio, 1.65; 95% confidence interval [CI], 1.21–2.25); however, changes in body weight were not related to type 2 diabetes risk (odds ratio, 0.95; 95% CI, 0.66–1.35).^[13]^ In another longitudinal cohort study, an increase of 1 SD in visceral fat in the L2 to L3 area (measured using CT) increased the risk of diabetes by 1.48 times (hazard ratio [HR], 1.48; 95% CI, 1.02–2.14) and by 1.79 times in the L4 to L5 area (HR, 1.79; 95% CI, 1.21–2.67); however, these relationships were only among men, and an increase in subcutaneous fat did not increase the risk of diabetes in either sex.^[11]^

Regarding its relationship with CVD, visceral fat increased the risk of CVD in a multiethnic cohort study with a 9.3-year follow-up. The likelihood of coronary heart disease was significantly higher among participants in the middle (HR, 2.43; 95% CI, 1.38–4.28) and highest (HR, 3.00; 95% CI, 1.66–5.43) tertiles than among those in the lowest tertile of visceral fat.^[10]^ A similar positive association between visceral fat and CVD was observed in a 5-year follow-up study based on the Framingham Heart Study (HR, 1.44; 95% CI, 1.08–1.92); however, there was no significant association between subcutaneous fat and CVD (HR, 0.99; 95% CI, 0.66–1.49).^[26]^

Based on these previous studies,^[7,11,26]^ central obesity, especially visceral fat, is strongly associated with metabolic and CVDs. Similar to these findings, we found that visceral fat indices such as VFA, VFM, and VFV predicted MS better than subcutaneous fat indices in both sexes. Considering that visceral fat is a crucial component in the development of MS, the cutoff values for VFA to determine MS have been proposed in several studies.^[21,27–29]^ In a large Korean population-based study, the cutoff values for VFA were 134.6 cm^2^ for men and 91.1 cm^2^ for women,^[21]^ which are relatively lower than those reported in our study, that is, 138 cm^2^ and 96.1 cm^2^ for men and women, respectively. While we determined the cutoff values to predict the presence of more than 3 metabolic risk factors, their cutoff values were used to predict 2 or more. This might explain the different VFA cutoff values. In another Korean study, the optimal cutoff values were 136 cm^2^ and 95 cm^2^ for men and women, respectively.^[28]^ These cutoff values are slightly lower than those in our study. Other authors used the criteria proposed by the International Diabetes Federation, whereas we used the Third National Cholesterol Education Program Expert Panel on Detection, Evaluation, and Treatment of High Blood Cholesterol in Adults criteria to define MS.

Abdominal obesity can be estimated using anthropometric measures, such as WC, waist-to-hip ratio, and waist-to-height ratio.^[14]^ However, these methods cannot directly measure the areas or volumes of adipose tissue. Furthermore, they cannot distinguish between visceral and subcutaneous fat.^[17]^ Contrary to anthropometric measures, CT and MRI can directly measure the areas or volumes of adipose tissue and distinguish between visceral and subcutaneous fat.^[14]^ Additionally, bioelectrical impedance analysis, ultrasonography, and dual-energy X-ray absorptiometry (DXA) can estimate visceral adiposity. However, there is no strong correlation between bioelectrical impedance and visceral fat, and the measurement of visceral fat via ultrasonography is not as accurate as that of WC.^[14]^ DXA-measured visceral adiposity has shown a strong correlation with MRI- or CT-measured visceral adiposity^[30]^; however, there are no longitudinal studies on whether DXA can detect visceral fat changes over time.^[14]^

CT is less expensive and less time-consuming than MRI, with fewer artifacts.^[17]^ Owing to radiation exposure and methodologic simplicity, single-slice CT is widely used to measure abdominal adiposity. However, VFA values obtained using single-slice CT can vary according to the patient's breathing rate and the area from where the image is obtained. As visceral fat can extend outward owing to the downward movements of the diaphragm on inspiration, it is crucial to measure VFA during late expiration.^[31]^ Therefore, single-slice CT imaging may not be as accurate as entire fat volume imaging,^[14]^ although it has been reported to be correlated with total VFV.^[31]^

In this study, we identified the optimal cutoff values for fat volumes to predict MS in a single ethnic group. To the best of our knowledge, such studies had been lacking prior to the report of a Japanese study in 2016.^[6]^ In that Japanese study, 405 participants were included, all of whom underwent health screening with CT. Similar to our study, VFV and SFV were calculated from the top of the liver to the pelvic floor using an automated software program. The VFV cutoff values were 3885 cm^3^ for men and 2321 cm^3^ for women, which are relatively lower than those reported in our study. The difference between our study and the Japanese study is that the Japanese authors determined the cutoff values to distinguish metabolically normal features from the presence of any MS feature, except WC, whereas we identified the cutoff values to predict MS by the presence of more than 3 metabolic risk factors. Moreover, the Japanese authors presented the cutoff values for VFV/height and VFV/abdominal length ratio, which had higher AUCs than VFV alone. Among those indices, the VFV/height ratio showed the greatest AUC for predicting risk factors for MS. Similar to these results, we found that the VFV/height ratio had the largest area in the ROC curve analysis, although the difference in the AUC between the VFV/height ratio and VFV alone was small and only observed in men (AUC of VFV/height ratio, 0.816 for men and 0.824 for women). In addition, the AUCs of VFV and VFM were higher in our study than in the Japanese study (0.811 for men and 0.826 for women in this study vs 0.746 for men and 0.762 for women in the Japanese study).

This study is the first to propose the cutoff values for VFV and VFM to predict MS in a Korean population. However, this study had some limitations. First, only relatively healthy people who underwent health screening examinations were included. The study population might not be representative of the general Korean population. Second, the actual prevalence of underlying diseases, such as hypertension or diabetes, was less likely to be accurately determined owing to the nature of the self-report questionnaire. Third, fat quantification using CT might not be applicable to the general population in real-world clinical settings, considering the high cost of the test and radiation exposure.

In conclusion, we suggest the optimal cutoff values for VFV and VFM to predict MS at 4852 cm^3^ and 4366.8 g and 3101 cm^3^ and 2790.9 g for men and women, respectively. Further large, population-based studies are needed to validate these cutoff values.

## Author contributions

**Conceptualization:** Yoon Jeong Cho.

**Data curation:** Yun-A Kim, Sang Gyu Kwak, Yoon Jeong Cho.

**Funding acquisition:** Yoon Jeong Cho

**Investigation:** Yun-A Kim, Yoon Jeong Cho.

**Project administration:** Yoon Jeong Cho.

**Resources:** Yun-A Kim, Yoon Jeong Cho

**Supervision:** Yoon Jeong Cho

**Writing – original draft:** Yun-A Kim.

**Writing – review & editing:** Yun-A Kim, Yoon Jeong Cho.

## References

[1] Alberti KG Eckel RH Grundy SM. Harmonizing the metabolic syndrome: a joint interim statement of the International Diabetes Federation Task Force on Epidemiology and Prevention; National Heart, Lung, and Blood Institute; American Heart Association; World Heart Federation; International Atherosclerosis Society; and International Association for the Study of Obesity. Circulation 2009;120:1640–5. 19805654 10.1161/CIRCULATIONAHA.109.192644

[2] Enkhmaa B Shiwaku K Anuurad E. Prevalence of the metabolic syndrome using the Third Report of the National Cholesterol Educational Program Expert Panel on Detection, Evaluation, and Treatment of High Blood Cholesterol in Adults (ATP III) and the modified ATP III definitions for Japanese and Mongolians. Clin Chim Acta 2005;352:105–13. 15653104 10.1016/j.cccn.2004.08.012

[3] Sato F Maeda N Yamada T. Association of epicardial, visceral, and subcutaneous fat with cardiometabolic diseases. Circ J 2018;82:502–8. 28954947 10.1253/circj.CJ-17-0820

[4] Cervantes A Singh RG Kim JU DeSouza SV Petrov MS. Relationship of anthropometric indices to abdominal body composition: a multi-ethnic New Zealand magnetic resonance imaging study. J Clin Med Res 2019;11:435–46. 31143311 10.14740/jocmr3820 PMC6522232

[5] Reijrink M de Boer SA Spoor DS. Visceral adipose tissue volume is associated with premature atherosclerosis in early type 2 diabetes mellitus independent of traditional risk factors. Atherosclerosis 2019;290:87–93. 31604171 10.1016/j.atherosclerosis.2019.09.016

[6] Tsukiyama H Nagai Y Matsubara F. Proposed cut-off values of the waist circumference for metabolic syndrome based on visceral fat volume in a Japanese population. J Diabetes Investig 2016;7:587–93. 10.1111/jdi.12454 PMC4931210 27181599

[7] Sam S. Differential effect of subcutaneous abdominal and visceral adipose tissue on cardiometabolic risk. Horm Mol Biol Clin Investig 2018;33:33. 10.1515/hmbci-2018-0014 29522417

[8] Jensen MD. Role of body fat distribution and the metabolic complications of obesity. J Clin Endocrinol Metab 2008;93:S57–63. 18987271 10.1210/jc.2008-1585 PMC2585758

[9] Bergman RN Ader M. Free fatty acids and pathogenesis of type 2 diabetes mellitus. Trends Endocrinol Metab 2000;11:351–6. 11042464 10.1016/s1043-2760(00)00323-4

[10] Mongraw-Chaffin M Allison MA Burke GL. CT-derived body fat distribution and incident cardiovascular disease: the multi-ethnic study of atherosclerosis. J Clin Endocrinol Metab 2017;102:4173–83. 28938406 10.1210/jc.2017-01113 PMC5673276

[11] Bray GA Jablonski KA Fujimoto WY. Relation of central adiposity and body mass index to the development of diabetes in the Diabetes Prevention Program. Am J Clin Nutr 2008;87:1212–8. 18469241 10.1093/ajcn/87.5.1212 PMC2517222

[12] Nicklas BJ Penninx BW Ryan AS Berman DM Lynch NA Dennis KE. Visceral adipose tissue cutoffs associated with metabolic risk factors for coronary heart disease in women. Diabetes Care 2003;26:1413–20. 12716798 10.2337/diacare.26.5.1413

[13] Wander PL Boyko EJ Leonetti DL McNeely MJ Kahn SE Fujimoto WY. Change in visceral adiposity independently predicts a greater risk of developing type 2 diabetes over 10 years in Japanese Americans. Diabetes Care 2013;36:289–93. 22966093 10.2337/dc12-0198 PMC3554282

[14] Fang H Berg E Cheng X Shen W. How to best assess abdominal obesity. Curr Opin Clin Nutr Metab Care 2018;21:360–5. 29916924 10.1097/MCO.0000000000000485 PMC6299450

[15] Nemoto M Yeernuer T Masutani Y. Development of automatic visceral fat volume calculation software for CT volume data. J Obes 2014;2014:495084. 24782922 10.1155/2014/495084 PMC3981487

[16] Villegas-Valle RC Lim U Maskarinec G. Metabolic syndrome screening using visceral adipose tissue (VAT) from opportunistic MRI locations in a multi-ethnic population. Obes Res Clin Pract 2021;15:227–34. 34024755 10.1016/j.orcp.2021.03.007

[17] Parikh AM Coletta AM Yu ZH. Development and validation of a rapid and robust method to determine visceral adipose tissue volume using computed tomography images. PLoS One 2017;12:e0183515. 28859115 10.1371/journal.pone.0183515 PMC5578607

[18] Okuno T Koseki K Nakanishi T. Prognostic impact of computed tomography-derived abdominal fat area on transcatheter aortic valve implantation. Circ J 2018;82:3082–9. 30298852 10.1253/circj.CJ-18-0709

[19] Murakami K Wada J Ogawa D. The effects of telmisartan treatment on the abdominal fat depot in patients with metabolic syndrome and essential hypertension: Abdominal fat Depot Intervention Program of Okayama (ADIPO). Diab Vasc Dis Res 2013;10:93–6. 22561230 10.1177/1479164112444640

[20] Ahn CW Kim CS Nam JH. Effects of growth hormone on insulin resistance and atherosclerotic risk factors in obese type 2 diabetic patients with poor glycaemic control. Clin Endocrinol (Oxf) 2006;64:444–9. 16584518 10.1111/j.1365-2265.2006.02490.x

[21] Kim JA Choi CJ Yum KS. Cut-off values of visceral fat area and waist circumference: diagnostic criteria for abdominal obesity in a Korean population. J Korean Med Sci 2006;21:1048–53. 17179685 10.3346/jkms.2006.21.6.1048 PMC2721927

[22] Choi MH Yoon SB Lee K. Preoperative sarcopenia and post-operative accelerated muscle loss negatively impact survival after resection of pancreatic cancer. J Cachexia Sarcopenia Muscle 2018;9:326–34. 29399990 10.1002/jcsm.12274 PMC5879976

[23] Furukawa K Katabami T Nakajima Y. Evaluation of whole-abdominal fat volume by 700-slice CT scanning and comparison with the umbilical fat area anthropometric indices. Obes Res Clin Pract 2010;4:e83–162. 10.1016/j.orcp.2009.10.001 24345649

[24] Obesity KSftSo. Clinical obesity. 3rd ed. Seoul: Korean Society for the Study of Obesity; 2008.

[25] Lee SH Tao S Kim HS. The prevalence of metabolic syndrome and its related risk complications among Koreans. Nutrients 2019;11:1755. 10.3390/nu11081755 PMC6724018 31366117

[26] Britton KA Massaro JM Murabito JM Kreger BE Hoffmann U Fox CS. Body fat distribution, incident cardiovascular disease, cancer, and all-cause mortality. J Am Coll Cardiol 2013;62:921–5. 23850922 10.1016/j.jacc.2013.06.027 PMC4142485

[27] Han JH Park HS Kim SM Lee SY Kim DJ Choi WH. Visceral adipose tissue as a predictor for metabolic risk factors in the Korean population. Diabet Med 2008;25:106–10. 18028439 10.1111/j.1464-5491.2007.02317.x

[28] Kim HI Kim JT Yu SH. Gender differences in diagnostic values of visceral fat area and waist circumference for predicting metabolic syndrome in Koreans. J Korean Med Sci 2011;26:906–13. 21738344 10.3346/jkms.2011.26.7.906 PMC3124721

[29] Oka R Kobayashi J Yagi K. Reassessment of the cutoff values of waist circumference and visceral fat area for identifying Japanese subjects at risk for the metabolic syndrome. Diabetes Res Clin Pract 2008;79:474–81. 18031862 10.1016/j.diabres.2007.10.016

[30] Cheung AS de Rooy C Hoermann R. Correlation of visceral adipose tissue measured by Lunar Prodigy dual X-ray absorptiometry with MRI and CT in older men. Int J Obes (Lond) 2016;40:1325–8. 27003112 10.1038/ijo.2016.50

[31] Ryo M Kishida K Nakamura T Yoshizumi T Funahashi T Shimomura I. Clinical significance of visceral adiposity assessed by computed tomography: a Japanese perspective. World J Radiol 2014;6:409–16. 25071881 10.4329/wjr.v6.i7.409 PMC4109092

